# Supplementary material for: Spatial and Temporal Diversity of Astrocyte Phenotypes in Spinocerebellar Ataxia Type 1 Mice
Source: Cells. 2022 Oct 21;11(20):3323. doi: 10.3390/cells11203323 (PMC9599982; doi:10.3390/cells11203323)
Supplement: Supplementary file 1 [file cells-11-03323-s001.zip › cells-1912036-supplementary.pdf]

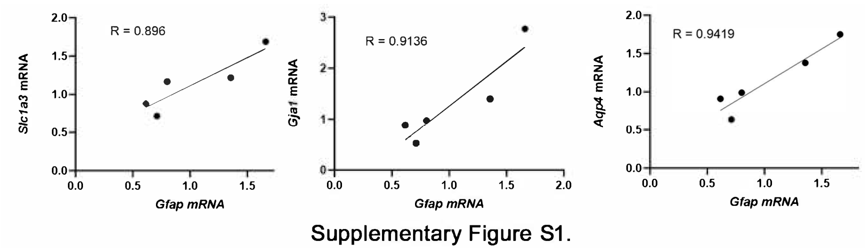

Supplementary Figure S1. **Pearson's correlation between Gfap and homeostatic astrocyte genes in the cerebellum.** mRNA was extracted from the cerebellum of 12 weeks old Atxn1154Q/2Q and wild-type littermate controls (N = 4-6) and RTqPCR was used to evaluate expression of astrocyte specific genes.

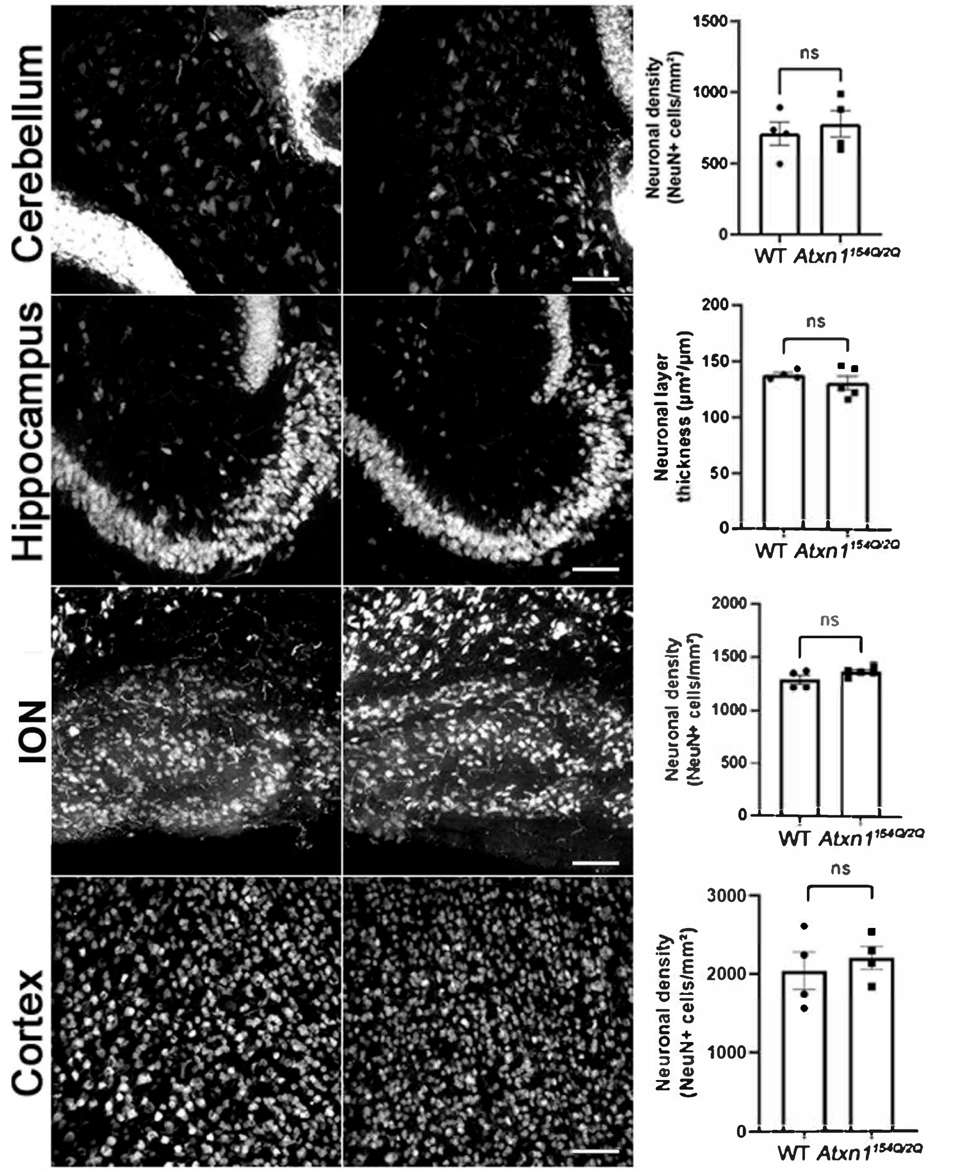

Supplementary Figure S2.

Supplementary Figure S2. **Neuronal density is not altered early in SCA1**. Brain sections from 12 weeks old Atxn1154Q/2Q and wild-type littermate controls (N = 3-5) were stained for NeuN. Confocal images of cerebellum (deep cerebellar nuclei), hippocampus (CA2/CA3), ION (inferior olivary nucleus), and motor cortex (layer 6) were used to quantify density of NeuN+ neurons. Scale bar is 100 µm. Data are presented as mean ± SEM with average values for each mouse represented by a dot. * $p<0.05$ Students t-test.

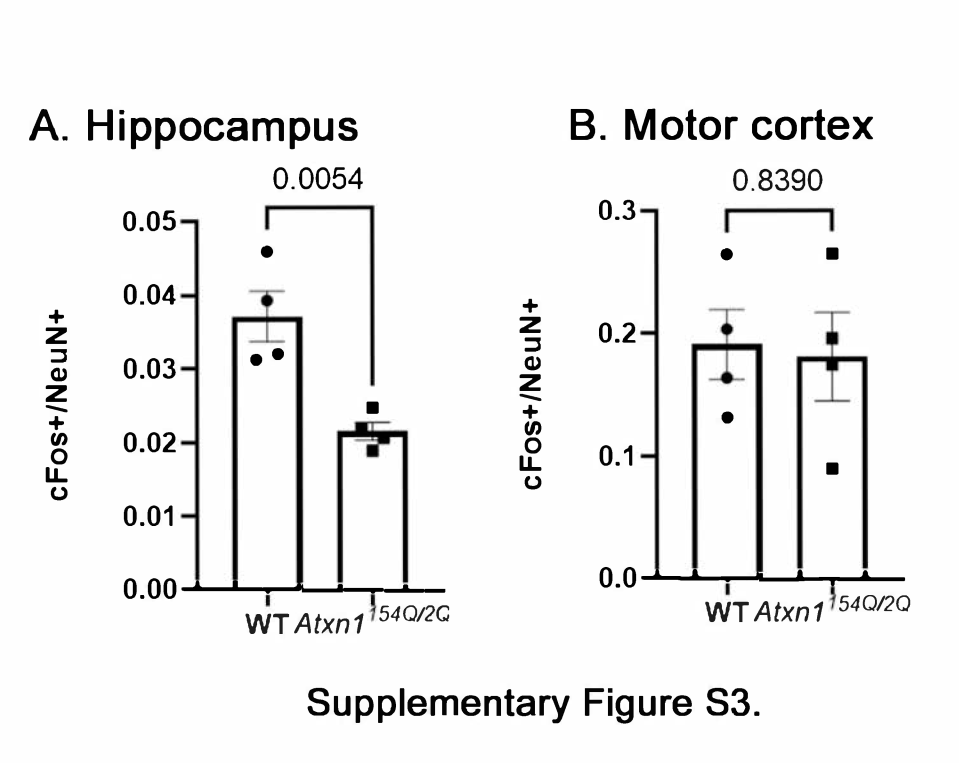

Supplementary Figure S3. **Neuronal activity is altered in the hippocampus but preserved in motor cortex early in SCA1.** Brain sections from 12 weeks old Atxn1154Q/2Q and wild-type littermate controls (N = 3-5) were stained for NeuN and c-Fos. Confocal images of hippocampus (A, dentate gyrus), and motor cortex (B, layer 6) were used to quantify density of NeuN+ and c-Fos+ neurons. Data are presented as a mean ratio of c-Fos/NeuN+ cells ± SEM with average values for each mouse represented by a dot. Student's t-test P values are shown.

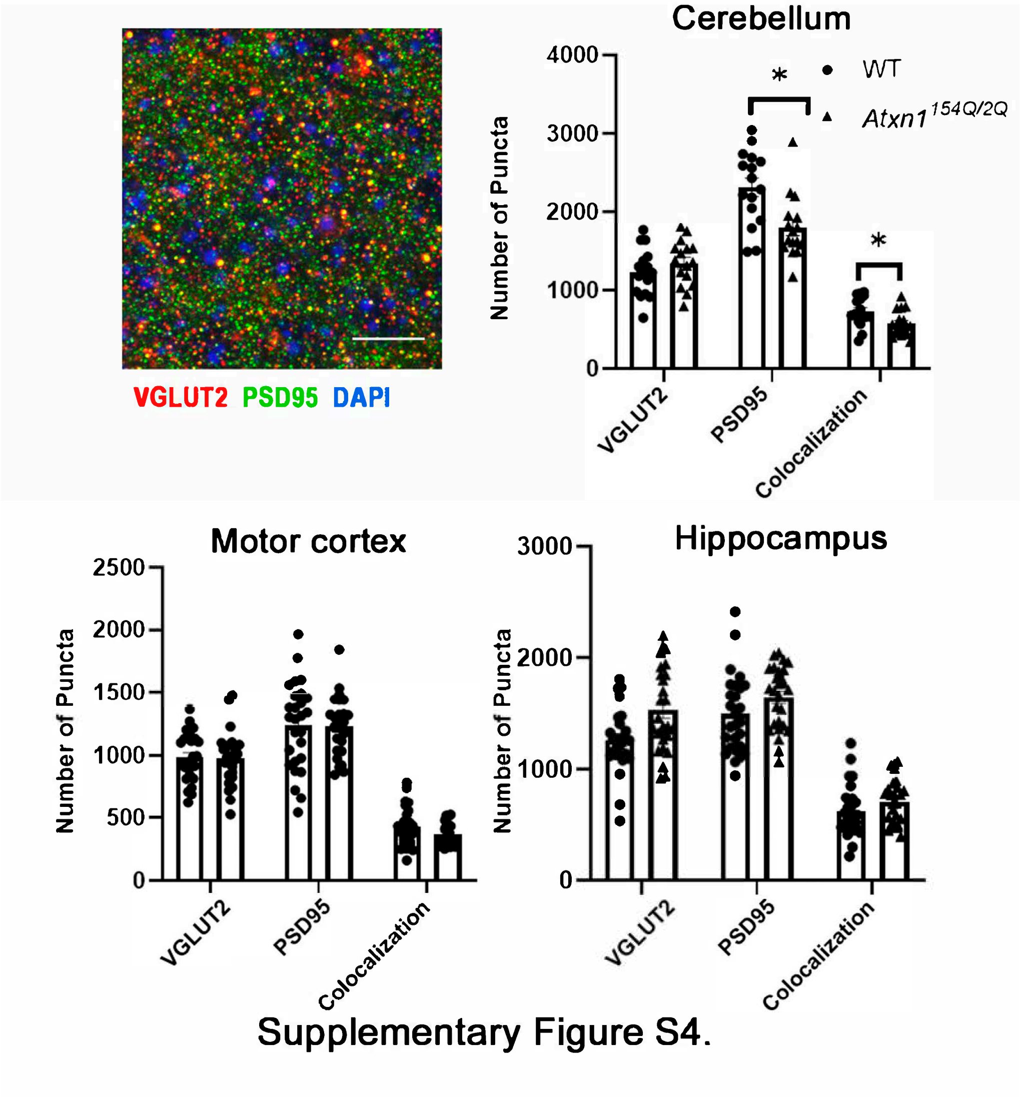

Supplementary Figure S4. Synaptic quantification in the cerebellum, hippocampus and cortex. Brain sections from 18 weeks old Atxn1154Q/2Q and wild-type littermate controls (N = 3-5) were stained for VGUT2 and PSD95. Confocal images were used to quantify the number of VGLUT2 and PSD 95 puncta and their overlay in cerebellum (lobule X upper 1/3), motor cortex and hippocampus (dentate gyrus). Scale bar is 10µm. Data are presented as a mean ± SEM with average values for each region of interest represented by a dot. * p<0.05 Students t-test.
